# Participants Attrition in a Longitudinal Study: The Malaysian Cohort Study Experience

**DOI:** 10.3390/ijerph18147216

**Published:** 2021-07-06

**Authors:** Noraidatulakma Abdullah, Mohd Arman Kamaruddin, Ying-Xian Goh, Raihannah Othman, Andri Dauni, Nazihah Abd Jalal, Nurul Ain Mhd Yusuf, Salywana A. Kamat, Nor Hazlinawati Basri, Rahman Jamal

**Affiliations:** UKM Medical Molecular Biology Institute (UMBI), Universiti Kebangsaan Malaysia, Kuala Lumpur 56000, Malaysia; noraidatulakma.abdullah@ppukm.ukm.edu.my (N.A.); arman@ppukm.ukm.edu.my (M.A.K.); yingxian@ukm.edu.my (Y.-X.G.); raihannah@ukm.edu.my (R.O.); andri@ppukm.ukm.edu.my (A.D.); nazihah.aj@ukm.edu.my (N.A.J.); ainmdy@ukm.edu.my (N.A.M.Y.); salywana@gmail.com (S.A.K.); lynnebasri@gmail.com (N.H.B.)

**Keywords:** cohort retention, attrition, the Malaysian Cohort, follow-up methodology, Asian population

## Abstract

The attrition rate of longitudinal study participation remains a challenge. To date, the Malaysian Cohort (TMC) study follow-up rate was only 42.7%. This study objective is to identify the cause of attrition among TMC participants and the measures to curb it. A total of 19,343 TMC participants from Kuala Lumpur and Selangor that was due for follow-up were studied. The two most common attrition reasons are undergoing medical treatment at another government or private health center (7.0%) and loss of interest in participating in the TMC project (5.1%). Those who were inclined to drop out were mostly Chinese, aged 50 years and above, unemployed, and had comorbidities during the baseline recruitment. We have also contacted 2183 participants for the home recruitment follow-up, and about 10.9% agreed to join. Home recruitment slightly improved the overall follow-up rate from 42.7% to 43.5% during the three-month study period.

## 1. Introduction

The challenges in a cohort study include the relatively high cost due to the prospective nature of the study [1], the labor-intensive follow-up process [2], and the drop in the response during follow-up [3]. The retention of participants is a big challenge, especially when the cohort participants are from the general population instead of a defined group of people with common traits [1]. Although the ideal follow-up rate of 50% is considered adequate, while 60% is good and 70% is very good [4], these numbers are difficult to achieve. During follow-up, the attrition rate of participants is crucial, as it might introduce a certain form of selection bias to the overall cohort study [5,6] and probably lead to the loss of statistical power in the analysis [7,8,9]. In addition, attrition can also be considered missing not at random (MNAR), which might influence the result of the analysis due to bias [10,11,12,13]. Since attrition could be a serious threat for longitudinal studies, previous studies have identified demographic and clinical characteristics associated with attrition. However, not all studies investigated the same factors, making it hard to summarize [14]. Some consistent findings related to attrition rate in longitudinal studies were aging, being a woman, fewer years of education, lower economic status, frailty, poor health, not having a family history of dementia, and cognitive impairment [14,15,16,17,18,19,20]. In addition, several neuropsychological tasks, such as having slower processing speed, less attentional flexibility, worse delayed verbal recall, and MRI markers, were associated with the attrition rate that might presumably be mediated by cognitive skills [16,21,22].

The Malaysian Cohort (TMC) project is a large population-based cohort study that managed to recruit 106,527 participants over a period from the year 2006 until 2012 [23]. The objective was to follow-up with the participants quinquennially (every five years) using the re-survey approach, a follow-up phone call, and passive surveillance to capture any morbidity and mortality events. During the re-survey, participants needed to answer a questionnaire and undergo biophysical measurements similar to the baseline recruitment plus other tests. Blood and urine samples were also collected. They were also called by phone to verify any morbidity and mortality event using a standardized set of questions. For passive surveillance, we received the mortality notification including the cause of death details every six months from the National Registry Department of Malaysia.

TMC follow-up methods [23] included a biannual health diary for participants to fill out and return, biannual follow-up calls, and a comprehensive quinquennial follow-up to retain the participants. Regular reminders were conducted via post mail, phone call, short messaging service, WhatsApp, non-monetary incentives, and tracing via family members (who are also participants), friends, and employers [3,24]. Despite all of these efforts, the attrition rate of 57.3% was still a major challenge. Thus, this paper aims to identify the cause of attrition among TMC participants and the measures to curb it.

## 2. Materials and Methods

### 2.1. Study Population

The study samples were those residing in the states of Kuala Lumpur and Selangor, which consisted of 41,696 participants. The following participants were then excluded: (1) 17,799 participants who had been successfully followed-up (42.7% follow-up rate), (2) 1458 participants (3.5%) who had died after baseline recruitment and verified by the National Registry Department of Malaysia as of December 2018, and (3) 3096 participants that had already agreed and scheduled a follow-up. The final number of participants in this follow-up study was 19,343. Written informed consent (read and signed) was obtained from the subjects. The project has been approved by the research ethics committee of the Universiti Kebangsaan Malaysia (Project code: FF-205-2007), in accordance with the Declaration of Helsinki.

### 2.2. Data Collection

There were two phases of the study: (1) follow-up appointment calls via phone using a standardized questionnaire and (2) home follow-up. In the first phase of the study, the data collection was done using a standardized computerized questionnaire that was filled out by trained staff members. The questionnaires ask information regarding the participants’ personal data, occupational history, self-reported medical history with medication, dietary habits, physical activity, and lifestyle histories, such as tobacco and alcohol use. Comorbidities, such as hypertension, hypercholesterolemia, diabetes mellitus, and obesity were measured using two methods: (1) self-reporting and (2) health assessment. For self-reported comorbidities, the participants were asked whether a medical doctor had diagnosed them with the disease and whether they had taken any medication for it. Among the diseases that were diagnosed through health assessment during the recruitment were hypertension (indicated by a blood pressure test was more than 140/90 mmHg), hypercholesterolemia (total cholesterol test was more than 5.2 mmol/L), diabetes mellitus (fasting blood glucose test was more than 7 mmol/L), and obesity (body mass index measurement was more than 30 kgm^−2^). The participants were each called for at least three attempts via their mobile number, home landline, or office telephone number recorded in the database. Their responses were then recorded and categorized into 22 common responses. Based on that, we identified the reasons that could be possibly resolved by doing a home follow-up, which involved our staff collecting data and performing health assessments at the participant’s residences. Among the 22 responses, participants with the following responses were selected for the home follow-up approach: (1) “The participant was uncertain to join for follow-up or made an ambiguous response”; (2) “The participant could not attend the follow-up due to work or just busy”; and (3) “The participant could not join due to transportation problems”. These three categories were selected because they were interested in joining, but were set back by their commitment and transportation problems. Thus, TMC takes the initiative to go to them for home follow-up. The home-follow-up approach was conducted for three months, from March 2019 until May 2019.

### 2.3. Preparation for Home Recruitment

The home follow-up team consisted of three members, including a driver, a phlebotomist who was also the on-site lab technician, and an enumerator who also did the biophysical measurements. Two teams were used during the study period, and each team was scheduled to visit four to five participants daily. A prefilled informed consent form and a shortened version of the questionnaire were printed and brought to the homes. The questionnaire was also prefilled with the previous data for the relevant participant to be cross-validated and updated during the home follow-up. The biospecimen tubes were already pre-labeled with the participant’s identification number (SID).

### 2.4. Home Recruitment

Anthropometric data (such as weight, height, and waist-hip circumference) were measured using a portable stadiometer (Harpenden, SERITEX, Wales, UK), a Seca weighing scale, and a Seca circumference measuring tape. Blood pressure measurements (both in the standing and sitting positions) were obtained using digital automatic blood pressure monitor (HEM-907, OMRON, Kyoto, Japan). The assessment was repeated three times, and the average value was recorded.

Blood and urine samples were collected using standard procedures. The blood-filled fluoride vacutainer was centrifuged on the spot and kept in a cool box immediately at 2–8 °C to prevent hemolysis. The samples were brought back to the laboratory at the main center for subsequent processing.

The questionnaire was administered via an interview and also recorded using a MP3 device. The whole interview session took about 15 min on average. The home follow-up process took an average of 35 to 40 min, excluding travel time. The time spent was notably shorter than the follow-up at our main recruitment center, which usually takes around two hours. The biophysical measurements and questionnaire data were recorded into the online database at the main center.

### 2.5. Statistical Analyses

All statistical analyses were performed using the Statistical Package for the Social Sciences (SPSS version 22.0, IBM, Armonk, NY, USA). Chi-squared tests calculated differences in appointment status based on gender, ethnicity, age group, education level, working status, and comorbidities, and a *p*-value of < 0.05 was considered statistically significant. Logistic regression using the likelihood ratio method was used to identify risk factors associated with attrition rate. Statistically significant risk factors identified in the uni-variable analysis (*p* < 0.05) were then included in the multivariable analysis. Odds ratios and their 95% confidence intervals were provided as estimates of the effect size, and the test was conducted at a 5% level of significance.

## 3. Results

### Participants’ Responses and Reasons of Attrition

The participants’ responses from the follow-up calls were described in Table 1. Out of 19,343 participants due for follow-up, 6.1% of calls were successful with participants agreeing to join; 0.2% had attended the follow up (44 people), 15.3% were unsuccessful due to participant’s withdrawal, 0.1% had died, while another 78.3% needed to be contacted again. Those considered as a dropout were those who withdrew from the study. The most frequent reasons stated by the abovementioned participants were that they already underwent treatment at another government or private health center (7.0%) and lost interest in participating in the TMC project (5.1%). Although we updated our mortality data from the National Registry Department of Malaysia biannually, we missed the 19 deaths in between.

The majority of the participants fell into the category that they needed to be contacted again (78.3%). The most frequent reasons for this category were unanswered telephone calls (23.1%) and those that could not make up their minds or uncertain about coming for follow-up (22.6%) (Table 1). Other reasons were related to participants’ commitments (4.4%), transportation problems (0.6%), issues with telephone numbers or calls (24.3%), and several other reasons that are listed in Table 1 (3.3%). Out of this 78.3%, 27.6% (5331) of participants in this category were suitable for home follow-up. They were those who were uncertain, busy, or with transportation problems. In addition, 2183 participants (34.2%) were selected based on their postcode areas and were asked whether they would agree to a home follow-up. A total of 86 participants agreed to come to the main center for follow-up while another 239 participants agreed to the home follow-up (10.9% of the 2183 selected participants). The new method of home follow-up successfully attracted participants to join the follow-up within the three months, although it only slightly improved the overall follow-up rate from 42.7% to 43.5%. Participants’ characteristics, such as gender, ethnicity, age, education level, employment status, and morbidity status, were significantly associated with the appointment call status, which fell into two categories: Ref. [1] successful appointment call, need to re-contact for an appointment, and [2] unsuccessful appointment call (Table 2). The characteristic for the latter was female (60.5%), Chinese (55%), aged 50–59 years old (38.2%), had a secondary education level (51.9%), unemployed (64.2%), obese (85.3%), and diabetic (82.8%) (Table 2).

In the crude analysis, participants who were Chinese, above 40 years of age, had a secondary level education, were unemployed, and suffering from hypertension, hypercholesterolemia, and obesity were significantly associated with unsuccessful appointment calls (*p*-value < 0.05 for all) (Table 3). However, in the final model, after adjustment for other risk factors, participants who were above 50 years old were more inclined to attrition (OR = 1.38 (95% CI = 1.08–1.77); OR = 1.78 (95% CI = 1.30–2.45)) compared to younger participants, while the Chinese had the highest tendency to attrition with an odds ratio of 2.50 (95% CI = 1.38–4.51) compared to other ethnic groups (Table 3). The odds of an unsuccessful call for follow-up were higher among participants who were unemployed compared to employed participants (OR = 1.20; 95% CI = 1.02–1.41). Comorbidities, such as hypertension (OR = 1.37, 95% CI = 1.18–1.59) and hypercholesterolemia (OR = 1.19, 95% CI = 1.02–1.39), were also associated with the unsuccessful appointment call.

## 4. Discussion

In this current study, our objective is to identify the cause of attrition among TMC participants and the measures to curb it. It is interesting to note that the most common withdrawal reasons based on this study were that the participants had already undergone medical treatment at another government or private healthcare center and the loss of interest to continue as a participant. Many of them were diagnosed with diabetes, hypertension, or hypercholesterolemia from the health assessment during the baseline recruitment and later underwent treatment at a government or private healthcare center. As the public healthcare service in Malaysia is still very subsidized under the government, the participants have easy access to treatment and follow-up services as patients [25,26]. For those who can afford or are covered by the employer’s healthcare benefits or their private healthcare insurance, the private healthcare center would be more convenient [25,27], thus leading to the attrition from our study. The participants might feel that they were already on a regular follow-up schedule for their illnesses, hence causing the loss of interest to further participate in the study. This was consistent with our findings that those not interested in joining a follow-up have comorbidities (87.5% out of 2913 participants). The other explanation could be that, during the baseline recruitment, the participants might have joined the study out of curiosity and incentives (such as the free health screening provided), which might have introduced the “expected utility” effect. Once this effect had worn off, the “expected utility” of joining the study would be overwhelmed by the effect of “expected cost” of follow-up (e.g., time and energy) [28], hence making the participants lose interest.

The significant factors contributing to the attrition rate in this study were Chinese ethnicity, above 50 years old, unemployed, and having comorbidities, such as hypertension and hypercholesterolemia. The withdrawal rate among Chinese people was higher than other ethnicities, such as Malay and Indian, which might be due to language barriers [29]. Various methods were implemented to solve this problem, such as providing questionnaires in several languages, such as English, Mandarin, and Tamil [23], to facilitate the participants’ understanding and using translator services to assist the participants during the follow-up recruitment and appointment call.

Our particular concern was those diagnosed with comorbidities at the baseline recruitment, which put them at a higher risk of developing complications in the future, yet were not interested in joining a follow-up. Another reason for this might be related to our results that most of the participants who were unable to be followed up with were unemployed and elderly, consistent with other studies [14,30,31,32,33]. It is a part of Asian culture that the children or the caregiver would take care of the elderly. This might lead to dependency on their children or caregivers, preventing them from going to the healthcare centers by themselves due to the increased frailty and transportation problems [12]. The elderly population in Malaysia is likely to be unemployed due to compulsory retirement, lack of skills, and health reasons [34,35]. Although there were some successful examples of the elderly cohort retention, the study was smaller (N = 693) and limited only to a defined rural area. To combat the attrition due to transportation limitations, we introduced the home follow-up as a possible solution.

During a short span of three months, the home follow-up was three times more successful (10.9% versus 3.9%) to attract follow-up compared to the conventional recruitment in which the participants were required to come to the main center. The overall follow-up rate increased by 0.8% (from 42.7% to 43.5%) after the home follow-up. If this applied to all of the 5331 potential participants (who agreed to home follow-up), it was projected that the follow-up rate would be improved to 44.9%. Although the improved percentage was still below the 50% cutoff point of an adequate follow-up rate as defined [4], the follow-up rate of more than 40% was still very acceptable [11].

This study has some limitations, including the fact that the contributing factors of attrition were inferred based on the baseline data. We do not know about the change of the participant’s information across the time, similar to those described previously [36]. In addition, this study only focused on the participants residing in Kuala Lumpur and Selangor, and does not represent the whole cohort study. Another limitation is that this study does not assess other important attrition risks, such as cognitive function and neuropsychological tasks.

## 5. Conclusions

Those who were Chinese, aged 50 years and above, unemployed, and had comorbidities during the baseline recruitment were more inclined towards an unsuccessful follow-up. The home follow-up approach is a useful solution, but will require some key resources, such as staff and travel time. Further investigation on the cost effectiveness, feasibility, and feedback from the participants would be needed before implementing it for a longer period.

## Figures and Tables

**Table 1:** Follow-up appointment calls responses of TMC participants residing in the Kuala Lumpur and Selangor area, Malaysia, March through May 2019 (N = 19,343).

Follow-Up Appointment Calls Responses	Frequency (%)
Successful appointment call:	1226 (6.3)
Participant agreed to come for follow-up	1182 (6.1)
Participants informed that they had attend the follow-up	44 (0.2)
Need to re-contact for an appointment via call or another platform:	15,125 (78.3)
The telephone call was not answered	4468 (23.1)
The participant was uncertain about coming/had an ambiguous response *	4375 (22.6)
The telephone number was not in service	2392 (12.4)
Wrong telephone number/changed to a different unknown number	1140 (5.9)
The participant unable to come due to busy/working *	849 (4.4)
The telephone number was not reachable	805 (4.2)
The participant requested to be called at another time	370 (1.9)
The participant unable to come due to being outside the recruitment area temporarily	261 (1.3)
Other reasons	148 (0.8)
Language barrier	109 (0.6)
The participant unable to come due to logistics problems *	107 (0.6)
The participant requested to change to another follow-up location (i.e., not at the main center)	73 (0.4)
The telephone call line was not clear	28 (0.1)
Unsuccessful appointment call:	2992 (15.4)
Withdrawal to participate in the study—reason: underwent treatment at another government or private health center	1363 (7.0)
Withdrawal to participate in the study—reason: no longer interested in the TMC project	991 (5.1)
The participant moved out to distant places from any TMC recruitment centers	332 (1.7)
Withdrawal to participate in the study—without giving any reason	209 (1.1)
The telephone number was not available in the database	59 (0.3)
The participant was deceased	19 (0.1)
Withdrawal to participate in the study—reason: disappointed with the TMC project	19 (0.1)

* Denotes potential for home recruitment follow-up.

**Table 2:** Baseline characteristics of TMC participants residing in the Kuala Lumpur and Selangor area according to appointment call scheme 2019. (N = 18,729).

Baseline Characteristics		Successful Appointment Call (N = 1196)	Need to Re-contact for Appointment (N = 14,620)	Unsuccessful Appointment Call (N = 2913)	Total Participants (N = 18,729)	*p*-Value
		N (%)	N (%)	N (%)	N (%)	
Gender	Male	516 (43.1)	5701 (39.0)	1190 (40.9)	7407 (39.5)	0.005 *
	Female	680 (56.9)	8919 (61.0)	1723 (59.1)	11,322 (60.5)	
Race	M’alay	326 (27.3)	4272 (29.2)	672 (23.1)	5270 (28.2)	<0.001 *
	Chinese	597 (49.9)	7798 (53.3)	1914 (65.7)	10,309 (55.0)	
	Indian	253 (21.2)	2367 (16.2)	300 (10.3)	2920 (15.6)	
	Others	20 (1.7)	183 (1.3)	27 (0.9)	230 (1.2)	
Age	<40	152 (12.7)	1698 (11.6)	242 (8.3)	2092 (11.2)	0.001 *
	40–49	518 (43.3)	6167 (42.2)	1107 (38.0)	7792 (41.6)	
	50–59	415 (34.7)	5129 (35.1)	1112 (38.2)	6656 (35.5)	
	>60	111 (9.3)	1626 (11.1)	452 (15.5)	2189 (11.7)	
Educational Level	Primary	244 (20.4)	3865 (26.4)	760 (26.1)	4869 (26.0)	<0.001 *
	Secondary	665 (55.6)	7641 (52.3)	1512 (51.9)	9818 (52.4)	
	Tertiary	287 (24.0)	3114 (21.3)	641 (22.0)	4042 (21.6)	
Employment status	Employed	335 (28.0)	4946 (33.8)	1043 (35.8)	6324 (33.8)	<0.001 *
	Unemployed	861 (72.0)	9674 (66.2)	1870 (64.2)	12,405 (66.2)	
Diabetes	No	184 (15.4)	2208 (15.1)	500 (17.2)	2892 (15.4)	0.019 *
	Yes	1012 (84.6)	12,412 (84.9)	2413 (82.8)	15,837 (84.6)	
Hypertension	No	504 (42.1)	6507 (44.5)	1512 (51.9)	8523 (45.5)	<0.001 *
	Yes	692 (57.9)	8113 (55.5)	1401 (48.1)	10,206 (54.5)	
Hypercholesterolemia	No	835 (69.8)	10,471 (71.6)	2183 (74.9)	13,489 (72.0)	<0.001 *
	Yes	361 (30.2)	4149 (28.4)	730 (25.1)	5240 (28.0)	
Obesity	No	209 (17.5)	2396 (16.4)	428 (14.7)	3033 (16.2)	0.035 *
	Yes	987 (82.5)	12,224 (83.6)	2485 (85.3)	15,696 (83.8)	

* Denotes a significant *p*-value at 0.05.

**Table 3:** Risk factors associated with attrition (unsuccessful) in TMC participants residing in the Kuala Lumpur and Selangor area, Malaysia, March through May 2019.

Baseline Characteristics	Unsuccessful Appointment Call	Crude Odds Ratio (95% CI)	*p*-Value	Adjusted Odds Ratio (95% CI)	*p*-Value
Gender	Male	1		-	
	Female	1.10 (0.96–1.26)	0.176		
Race	Others	1		1	
	Malay	1.53 (0.84–2.76)	0.162	1.66 (0.91–3.02)	0.098
	Chinese	2.37 (1.32–4.26)	0.004 *	2.50 (1.38–4.51)	0.002 *
	Indian	0.88 (0.48–1.60)	0.673	0.91 (0.50–1.67)	0.760
Age	<40	1		1	
	40–49	1.34 (1.07–1.69)	0.011 *	1.23 (0.97–1.55)	0.088
	50–59	1.68 (1.33–2.12)	<0.001 *	1.38 (1.08–1.77)	0.009 *
	>60	2.56 (1.91–3.42)	<0.001 *	1.78 (1.30–2.45)	<0.001 *
Educational Level	Primary	1		–	
	Secondary	0.73 (0.62–0.87)	0.001 *		
	Tertiary	0.72 (0.59–0.88)	0.833		
Working status	Employed	1		1	
	Unemployed	1.43 (1.24–1.66)	<0.001 *	1.20 (1.02–1.41)	0.026 *
Diabetes	No	1		–	
	Yes	1.14 (0.95–1.37)	0.164		
Hypertension	No	1		1	
	Yes	1.48 (1.29–1.70)	<0.001 *	1.37 (1.18–1.59)	<0.001 *
Hypercholesterolemia	No	1		1	
	Yes	1.29 (1.11–1.50)	0.001 *	1.19 (1.02–1.39)	0.030*
Obesity	No	1		–	
	Yes	0.81 (0.68–0.97)	0.025 *		

* Denotes a significant *p*-value at 0.05.

## Data Availability

The datasets used and analyzed during the current study are available from the corresponding author on reasonable request.
